# Comparison of the methods for detecting lower limb asymmetries: isokinetic dynamometry vs. tensiomyography derived indices

**DOI:** 10.3389/fspor.2026.1793309

**Published:** 2026-03-27

**Authors:** Lazar Toskić, Armin Paravlić, Radenko Arsenijević, Nikola Aksović, Ivan Čurović, Milan Marković, Saša Bubanj, Milivoj Dopsaj

**Affiliations:** 1 Faculty of Sport and Physical Education, University of Priština in Kosovska Mitrovica, Leposavić, Serbia; 2 Faculty of Sport, University “Union-Nikola Tesla”, Belgrade, Serbia; 3 Faculty of Sport, University of Ljubljana, Ljubljana, Slovenia; 4 Scientific Research Center Koper, Institute for Kinesiology Research, Koper, Slovenia; 5 Faculty of Sports Studies, Incubator of Kinanthropology Research, Masaryk University, Brno, Czechia; 6 Institute of Coaching and Performance, University of Central Lancashire, Preston, United Kingdom; 7 Faculty of Sport and Physical Education, University of Niš, Niš, Serbia; 8 Faculty of Sport and Physical Education, University of Belgrade, Belgrade, Serbia

**Keywords:** muscle asymmetry, tensiomyography, isokinetic dynamometry, correlations, sex

## Abstract

**Introduction:**

The purpose of this study was to investigate the relationships between asymmetry indices derived from isokinetic dynamometry (ID) and tensiomyography (TMG).

**Methods:**

Adult males and females (*N* = 158, Age = 23.7 ± 3.18 years) were recruited from three cohorts: physically inactive, physically active non-athletes, and professional athletes. Both ID (60°/s −60 and 180°/s −180) and TMG were applied to the knee joint extensor (Q) and flexor (H) muscles of the right (R) and left leg (L), after which functional (FS) and lateral asymmetry (LS) indices were calculated.

**Results and Discussion:**

Pearson correlation revealed no significant correlations between ID and TMG in FS. For LS, significantly small-to-moderate negative correlations were found between LS H180, LS of the Semitendinosus muscle and LS Havg in males (r = −0.337, *p* = 0.002; r = −0.322, *p* = 0.003, respectively). The present findings indicate that ID and TMG measure different dimensions of muscle asymmetry and therefore should not be considered interchangeable. While TMG provides valuable information on peripheral contractile properties, it does not appear to replicate the joint-level asymmetry outcomes obtained with ID, and thus may not serve as a direct substitute for isokinetic assessment when evaluating lower-limb muscle asymmetries.

## 1 Introduction

Lateral preference and sport-specific loading drive side-to-side differences: most individuals favour one (1), and repeated unilateral actions—as in kicking or jumping, exacerbate these imbalances (2). In the context of knee musculature, asymmetries exceeding 10% in peak torque have been linked to increased injury risk and impaired force production (3), yet precise quantification remains method- and population-dependent (4–6).

There are numerous methods for the assessment of muscle asymmetry. Isokinetic dynamometry (ID) is a method of testing muscle strength using a machine that maintains a constant speed of movement while measuring the applied force. It is widely used for quantifying knee-extension and knee-flexion torque at fixed angular velocities (e.g., 60°/s and 180°/s) and for assessing both lateral (inter-limb) and functional (intra-limb) asymmetries (7, 8). Isokinetic assessment has been recognized as an important tool for both training and rehabilitation optimization. Contemporary return-to-sport guidelines for lower-limb injuries, such as anterior cruciate ligament and its reconstruction, highlight the method's clinical value and recommend that athletes achieve up to 100% limb-to-limb symmetry before resuming high-demand pivoting sports (9). Because these tests depend on the participant's voluntary maximal effort, the results are partly influenced by motivation (10), which may affect its practical utility. Unlike with ID, tensiomyography (TMG) measures a muscle's passive response to an electrical stimulus to assess its intrinsic contractile properties, such as contraction time (Tc) and maximal displacement (Dm), rather than measuring the force output of a voluntary maximal contraction (11, 12). Because TMG is less influenced by participant effort or motivation, it has the potential to serve as a low-effort proxy for detecting muscle asymmetries (13). Additionally, TMG equipment is portable, which makes it more applicable in different environments than ID.

To date, only two studies have explored the relationship between asymmetries measured by ID and TMG (14, 15) and found conflicting results. In elite female soccer players, the authors found a positive association between functional asymmetries of the knee-joint muscles assessed with TMG and knee-flexion torque asymmetry at 60°/s (14). By contrast, lateral asymmetries of individual muscles—such as the Vastus medialis contraction time (Tc) and the Rectus femoris contraction velocity (Vc)—were negatively correlated with functional asymmetry at both 60°/s and 180°/s, and with knee-extension torque asymmetry at 180°/s (14). Additionally, weak to moderate negative correlations were observed between functional isokinetic knee muscle ratios and TMG ratios of contraction time and delay time (15). These findings underscore the intricate interplay between the contractile characteristics of individual quadriceps and hamstring heads and the torque metrics derived from ID.

Most extant asymmetry research has involved small homogeneous cohorts—often male team-sport athletes or track and field athletes—limiting generalizability (16). Yet adult individuals span a spectrum of activity backgrounds, from inactive through recreationally active to specialized power, endurance or team-sport athletes. Also, sex differences in muscle architecture and neuromuscular recruitment further complicate the picture (17). To date, no study has directly compared functional and lateral asymmetry indices from ID and TMG within a single, large heterogeneous sample that includes both males and females across varied activity profiles. By directly comparing mechanical (torque) and contractile (TMG) measures of muscle balance in a diverse cohort, we aim to determine whether TMG can reliably detect muscle imbalances captured with the widely used ID—and thus offer a rapid, low-effort tool for guiding training and injury-prevention strategies in both male and female athletes of all activity levels.

Accordingly, this investigation will recruit adult participants of both sexes drawn from three activity cohorts, including inactive, recreationally active, and professional athletes (strength and power, endurance, and team sport), respectively. We aimed to: (1) quantify functional and lateral asymmetries using ID at 60°/s and 180°/s for knee joint extension and flexion, alongside TMG-derived measures for quadriceps and hamstrings; and (2) examine the associations between these asymmetry indices across sexes.

## 2 Materials and methods

### 2.1 Sample of participants

The sample of participants consisted of 158 individuals, 83 males (Age = 24.08 ± 3.74 years, Body height = 182.1 ± 7.06 cm, Body weight = 79.6 ± 7.06 kg) and 75 females (Age = 23.5 ± 2.62 years, Body height = 168.4 ± 6.92 cm, Body weight = 60.2 ± 8.02 kg). Participants had different training backgrounds, including physically inactive individuals (not involved in regular daily physical activities), physically active non-athletes (3–5 times per week, moderate to intensive physical activities), and professional athletes from different sports groups: strength and power athletes, endurance athletes, teams sports athletes (track and field, cycling, swimming, judo, wrestling, karate, football, basketball, volleyball, handball, water polo). The exclusion criteria for every participant were the presence of musculoskeletal injury in the past 6 months or illness in the past two weeks that could influence their performance.

All of the participants were healthy, rested, acquainted with the goals and risks of the research, and the testing included individuals who had voluntarily agreed to participate in the research and who signed an institutionally approved informed consent document. All of the testing was done in accordance with the Declaration of Helsinki and the rules of the Ethics Committee of the Faculty of Sport and Physical Education (IRB: 484-2).

### 2.2 Procedures

The participants were first measured with TMG, without a warm-up, and then with ID, which was preceded by a warm-up protocol. Before measurement, participants were familiarised with the protocol. All measurements were performed under the same conditions. The participants were tested in the morning, having been well rested and not engaged in physical activity in the 24 h preceding the test. All tests were performed by a team of researchers, which included three experienced personnel. All measurements were performed in the Methodology-research laboratory of the Faculty of Sport and Physical Education.

The ID evaluation was carried out on a Kin-Com AP125 isokinetic dynamometer (Chattecx Corp., Chattanooga, TN, USA). Measurements were done on the knee joint extensor (Q) and flexor (H) muscles of the right (R) and left (L) leg in concentric mode at velocities of 60 (60) and 180 (180)˚/s (18, 19). Before the measurement, the participants were required to warm up for a period of approximately 10 min (5 min on a stationary bicycle and 5 min of static leg muscle stretching) to prevent injury and maximise performance (19–21). The participants were seated in an upright position and fixed to the testing apparatus with the straps around the pelvis, thigh and malleoli and from that position, they performed the near maximum extension in the knee joint, starting with the flexed knee at an angle of 90˚, and then reverse flexion to the initial position. Each participant performed 5 repetitions at both speeds, with the first testing carried out at a lower speed (60 ˚/s). The rest between the sets was 3 min (19). The representative isokinetic parameter was the peak torque (PT). Functional (FS) and lateral (LS) asymmetry was calculated following previous studies (5, 8, 22).

Functional asymmetry (H/Q ratio) was calculated as follows: *PT H/PT Q*. Lateral asymmetry was calculated as follows: 100/(maxvalue)*(minvalue)*(-1)+100.

An ‘IF function' was added to the end of the formula in Microsoft Excel to indicate inter-limb differences individually: *IF (left *<* right, 1, −1), which ensured that the magnitude of asymmetry was not altered when different limbs performed in a superior manner (22).

TMG was applied on the superficial muscles around the knee joint, Rectus femoris (RF), Vastus medialis (VM), Vastus lateralis (VL), Biceps femoris (BF) and Semitendinosus (ST). Following established protocols (3, 11, 12, 14), in supine (RF, VM, VL) and prone (BF, ST) position, an isometric twitch was elicited via electrical stimulation and the resulting radial displacement of the muscle belly was captured at the skin surface by a TMG-BMC digital displacement sensor (Ljubljana, Slovenia). Detailed testing procedures have been described elsewhere (11, 12, 14, 19).

Based on those values, the algorithm proposed by the TMG and previous studies (14, 23, 24) for calculating both the lateral and functional asymmetries was implemented (Equations 1, 2).

Equation 1:

$$\mathrm{LS}=0.1\times \;\frac{\mathrm{MIN}(\mathrm{TdR};\mathrm{TdL})}{\mathrm{MAX}(\mathrm{TdR};\mathrm{TdL})}+0.6\times \frac{\mathrm{MIN}(\mathrm{TcR};\mathrm{TcL})}{\mathrm{MAX}(\mathrm{TcR};\mathrm{TcL})}+0.1\times \frac{\mathrm{MIN}(\mathrm{TsR};\mathrm{TsL})}{\mathrm{MAX}(\mathrm{TsR};\mathrm{TsL})}+0.2\times \;\frac{\mathrm{MIN}(\mathrm{DmR};\mathrm{DmL})}{\mathrm{MAX}(\mathrm{DmR};\mathrm{DmL})}\times 100$$

where LS represents the lateral asymmetry, MIN—the minimum, MAX—the maximum, R—right leg parameters and L—left leg parameters.

Equation 2:

$$\mathrm{FS}=0.1\;\times \;\frac{\mathrm{MIN}(\mathrm{AVERAGE}(\mathrm{TdRF};\mathrm{TdVL};\mathrm{TdVM});\mathrm{TdBF})}{\mathrm{MAX}(\mathrm{AVERAGE}(\mathrm{TdRF};\mathrm{TdVL};\mathrm{TdVM});\mathrm{TdBF})}+0.8\times \;\frac{\mathrm{MIN}(\mathrm{AVERAGE}(\mathrm{TcRF};\mathrm{TcVL};\mathrm{TcVM});\mathrm{TcBF})}{\mathrm{MAX}(\mathrm{AVERAGE}(\mathrm{TcRF};\mathrm{TcVL};\mathrm{TcVM});\mathrm{TcBF})}+0.1\;\times \;\frac{\mathrm{MIN}(\mathrm{AVERAGE}(\mathrm{TrRF};\mathrm{TrVL};\mathrm{TrVM});\mathrm{TrBF})}{\mathrm{MAX}(\mathrm{AVERAGE}(\mathrm{TrRF};\mathrm{TrVL};\mathrm{TrVM});\mathrm{TrBF})}\;\times 100$$

where FS represents a functional asymmetry, MIN—the minimum, MAX—the maximum, R—right leg parameters and L—left leg parameters.

Additionally, the average values of asymmetry for all muscles regarding the knee extensor (RF, VM, VL—LS Qavg) and flexor muscles (BF, ST—LS Havg) were calculated.

### 2.3 Statistical analysis

The Shapiro–Wilk's test was employed to assess the normality of the distribution, and all data sets confirmed normal distribution (*p* > 0.05). To examine the relationship between ID and TMG dependent variables, Pearson's correlation coefficient was employed. The strength of the Pearson correlation coefficient (r) was used by recommendations of Hopkins et al. (25), and categorized as: small (0.1), moderate (0.3), large (0.5), very large (0.7), and extremely large (0.9). To adjust for family-wise error in a case of multiple performed correlations, the Bonferroni adjustment has been applied (26). Where the significance was established, scatter plots were provided. The statistical analysis was performed using IBM SPSS Statistics software package (version 25, SPSS Inc., Chicago, IL, USA), and Microsoft Office Excel 2024 (Microsoft Corporation, Redmond, WA, USA). All variables are presented as mean and standard deviation.

## 3 Results

Means and standard deviations for FS and LS variables assessed using ID and TMG methods are displayed in Tables 1, 2. It can be noticed that both FS and LS values are lower when measured at lower velocities with ID, in males and females (1.033% vs. 0.981, on average, Table 1). Regarding TMG, higher values of FS are detected in the left leg in males (77.7%) and the right (73.6%) leg in females. The knee joint extensor muscles have higher symmetry values on average (84.4%), the VM muscle showed the highest values of LS (85%), while the ST muscle exhibits the lowest values (77.5%) (Table 2) in both sexes.

**Table 1:** Descriptive statistics values of functional and lateral asymmetry assessed with isokinetic dynamometry.

Variables	Male		Female	
	Mean	SD	Mean	SD
H/Q R60 (%)	0.644	0.090	0.642	0.102
H/Q R180 (%)	0.716	0.094	0.728	0.117
H/Q L60 (%)	0.651	0.082	0.647	0.091
H/Q L180 (%)	0.720	0.091	0.741	0.107
LS Q60 (%)	2.874	9.700	1.082	8.320
LS Q180 (%)	2.289	8.864	1.321	9.041
LS H60 (%)	1.643	10.273	0.087	9.591
LS H180 (%)	1.839	9.097	−0.501	9.464

H/Q R60, hamstring-quadriceps index for right leg at velocity of 60°/s; H/Q R180, hamstring-quadriceps index for right leg at velocity of 180°/s; H/Q L60, hamstring-quadriceps index for left leg at velocity of 60°/s; H/Q L180, hamstring-quadriceps index for left leg at velocity of 180°/s; LS Q60, lateral asymmetry of the knee joint extensor muscles measured at velocity of 60°/s; LS Q180, lateral asymmetry of the knee joint extensor muscles measured at velocity of 180°/s; LS H60, lateral asymmetry of the knee joint flexor muscles measured at velocity of 60°/s; LS H180, lateral asymmetry of the knee joint flexor muscles measured at velocity of 180°/s.

**Table 2:** Descriptive statistics values of functional and lateral asymmetry assessed with tensiomyography.

Variables	Male		Female	
	Mean	SD	Mean	SD
FS R (%)	77.5	10.8	73.6	11.2
FS L (%)	77.7	11.7	72.6	11.6
LS RF (%)	83.4	7.3	85.4	6.5
LS VL (%)	84.9	7.2	86.4	7.9
LS VM (%)	85.0	7.3	85.9	8.6
LS Qavg (%)	84.4	4.3	85.9	4.7
LS BF (%)	81.5	10.6	84.4	8.4
LS ST (%)	77.5	14.2	76.8	13.08
LS Havg (%)	79.5	9.1	80.7	8.6

FS R, functional asymmetry for right leg; FS L, functional asymmetry for left leg; LS RF, lateral asymmetry of the Rectus femoris muscle; LS VL, lateral asymmetry of the Vastus lateralis muscle; LS VM, lateral asymmetry of the Vastus medialis muscle; LS Qavg, average values of the knee joint extensor muscles lateral aysmmetry; LS BF, lateral asymmetry of the Biceps femoris muscle; LS ST, lateral asymmetry of the Semitendinosus muscle; LS Havg, average values of the knee joint flexor muscles lateral asymmetry.

Based on the results in Table 3, which represent the results of Pearson's correlation coefficient, it can be concluded that there was no established significant correlation between variables that monitored FS using an ID (H/Q R60, H/Q R180, H/Q L60, H/Q L180) and TMG (FS R and FS L) in both male and female sample (Bonferroni adjusted significance was set at *p* < 0.006).

**Table 3:** Correlation between functional asymmetry assessed with isokinetic dynamometry and tensiomyography.

Variables	H/Q R60		H/Q R180			H/Q L60		H/Q L180	
	r	p	r	p		r	p	r	p
Male									
FS R	−0.090	0.415	−0.093	0.402	FS L	0.062	0.575	0.171	0.119
Female									
FS R	0.010	0.935	−0.035	0.767	LS L	0.162	0.168	0.074	0.532

H/Q R60, isokinetic dynamometry hamstring-quadriceps index for right leg at velocity of 60°/s; H/Q R180, isokinetic dynamometry hamstring-quadriceps index for right leg at velocity of 180°/s; H/Q L60, isokinetic dynamometry hamstring-quadriceps index for left leg at velocity of 60°/s; H/Q L180, hamstring-quadriceps index for left leg at velocity of 180°/s; FS R, functional asymmetry for right leg measured by the TMG; FS L, functional asymmetry for left leg measured by the TMG, r, correlation coefficient, p, statistical significance, Bonferroni adjustment significance level *p* < 0.006.

**Figure 1:**
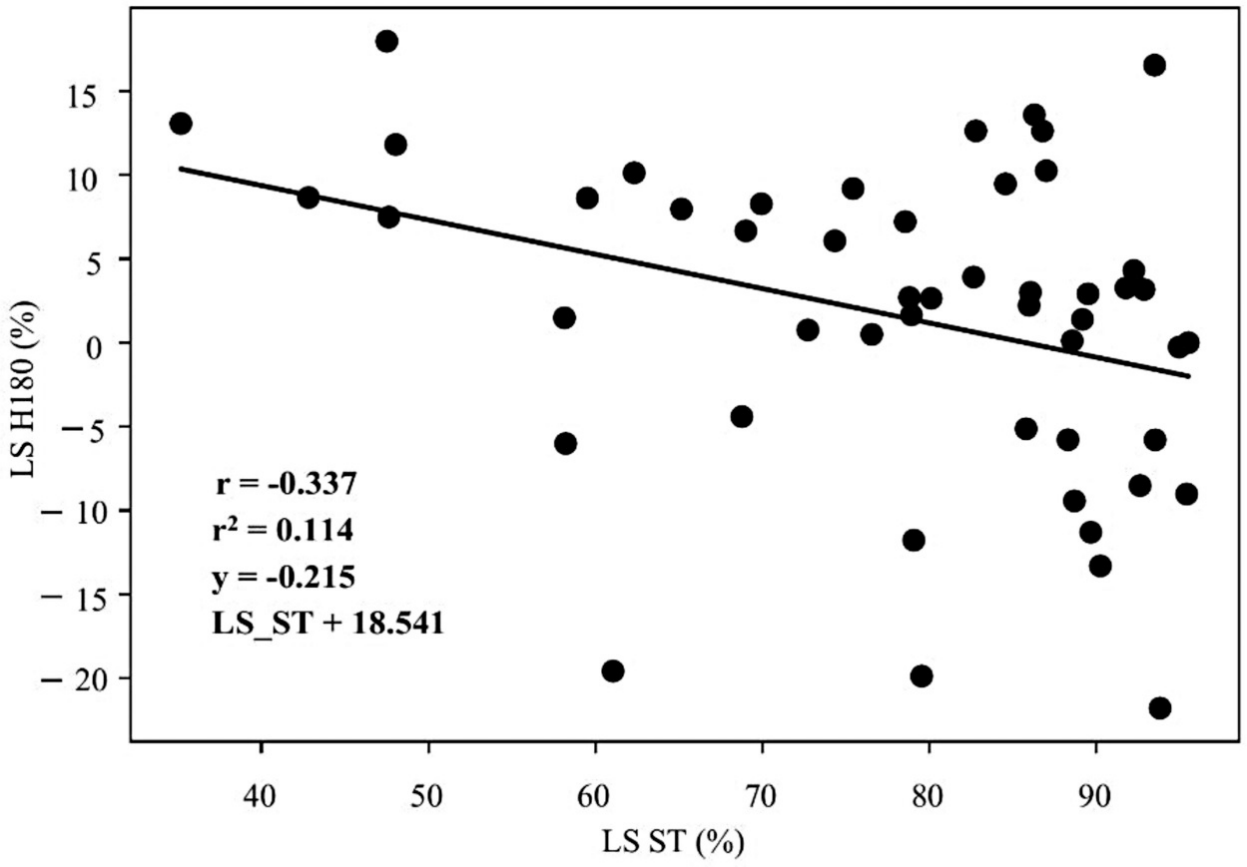
Results of correlation coefficient (r), coefficient of determination (r^2^) and its equation between the lateral asymmetry of the ST muscle (TMG, LS ST, *x*-axis) and the knee joint flexor muscles measured at a velocity of 180°/s (ID, LS H180, *y*-axis), for males.

**Figure 2:**
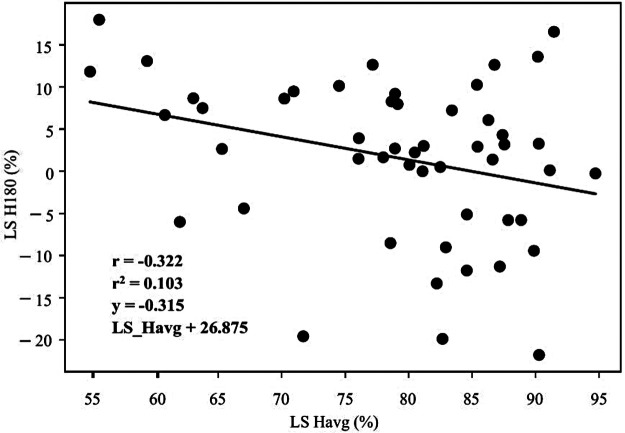
Results of correlation coefficient (r), coefficient of determination (r^2^) and its equation between the lateral asymmetry of the knee joint flexor muscles average value (TMG, LS havg, *x*-axis) and lateral asymmetry of the knee joint flexor muscles measured at a velocity of 180°/s (ID, LS H180, *y*-axis), for males.

Tables 4, 5 represent the results of Pearson's correlation between the LS assessed through ID and TMG. The obtained variables of the knee joint extensor muscles have stayed out of significance (Table 4, Bonferroni adjusted significance was set at *p* < 0.003). Regarding the knee joint flexor muscles, moderate negative correlation was observed between LS H180, LS ST and LS Havg in males (r = −0.337, *p* = 0.002; r = −0.322, *p* = 0.003, respectively, Table 5, Figures 1, 2, Bonferroni adjusted significance was set at *p* < 0.004).

**Table 4:** Correlation between the lateral asymmetry of the knee joint extensor muscles assessed with isokinetic dynamometry and tensiomyography.

Variables	LS RF		LS VL		LS VM		LS Qavg	
	r	*p*	r	*p*	r	*p*	r	*p*
Male								
LS Q60	0.016	0.884	−0.068	0.536	0.008	0.944	−0.026	0.814
LS Q180	−0.017	0.879	−0.190	0.084	−0.077	0.486	−0.177	0.107
Female								
LS Q60	−0.052	0.661	−0.205	0.079	−0.159	0.175	−0.233	0.046
LS Q180	0.136	0.253	−0.084	0.475	−0.049	0.677	−0.016	0.890

LS Q60, isokinetic dynamometry lateral asymmetry of the knee joint extensor muscles measured at velocity of 60°/s; LS Q180, isokinetic dynamometry lateral asymmetry of the knee joint extensor muscles measured at velocity of 180°/s; LS RF, lateral asymmetry of the Rectus femoris muscle measured by the TMG; LS VL, lateral asymmetry of the Vastus lateralis muscle measured by the TMG; LS VM, lateral asymmetry of the Vastus medialis muscle measured by the TMG; LS Qavg, average values of the knee joint extensor muscles lateral aysmmetry measured by the TMG; r, correlation coefficient, p, statistical significance, Bonferroni adjustment significance level *p* < 0.003.

**Table 5:** Correlation between the lateral asymmetry of the knee joint flexor muscles assessed with isokinetic dynamometry and tensiomyography.

Variables	LS BF		LS ST		LS Havg	
	r	*p*	r	*p*	r	*p*
Male						
LS H60	−0.188	0.087	−0.087	0.434	−0.186	0.091
LS H180	−0.102	0.356	**−0**.**337**	**0**.002**	**−0**.**322**	**0**.003**
Female						
LS H60	−0.014	0.904	−0.076	0.521	−0.062	0.602
LS H180	−0.022	0.856	−0.001	0.992	−0.006	0.963

LS H60, isokinetic dynamometry lateral asymmetry of the knee joint flexor muscles measured at velocity of 60°/s; LS H180, isokinetic dynamometry lateral asymmetry of the knee joint flexor muscles measured at velocity of 180°/s; LS BF, lateral asymmetry of the Biceps femoris muscle measured by the TMG; LS ST, lateral asymmetry of the Semitendinosus muscle measured by the TMG; LS Havg, average values of the knee joint flexor muscles lateral asymmetry measured by the TMG; r, correlation coefficient, r, correlation coefficient, p, statistical significance.

** Bonferroni adjustment significance level *p* < 0.004.

Bold values are significant values.

## 4 Discussion

This study aimed to compare the ID and TMG as methods for the assessment of lower-limb muscle asymmetry. To the best of the authors’ knowledge, this is the first study that directly compared these two modalities for lower-limb muscle asymmetry in both males and females and different PA background, addressing a critical gap in the literature. The results provide important information about the construct divergence analysis of TMG against the widely used ID, contributing valuable insights for sports diagnostics, performance optimization, and injury prevention. The main finding of this study is that only significant correlation was detected between LS H180 (ID), LS ST and LS Havg (TMG) in males (Table 5 and Figures 1, 2). These results are in accordance with previous research, which showed low and inconsistent correlation between muscle asymmetry assessed through ID and TMG (14). Specifically, a study comparing isokinetic torque ratios and TMG parameters in knee muscles of professional female soccer players found that most correlations were not statistically significant, ranging from very weak to moderate, with only weak to moderate negative correlations observed for specific functional isokinetic knee muscle ratios and TMG contraction and velocity times (14). Similar results were confirmed in healthy young adults, where only weak to moderate negative correlations between functional isokinetic knee muscle ratios and TMG ratios of contraction time and delay time were statistically significant (15).

These findings are, to some extent, expected and can be explained by the fundamental differences in the nature of ID and TMG as methods for measuring muscle characteristics and muscle asymmetry. Namely, ID and TMG are structurally very different methods, which have different measuring approaches and assess different muscle properties. While ID measurements are based on the movement and active, voluntary participation of the subject, capturing dynamic muscular function and integrated neural and muscular output, TMG is a non-voluntary method that assesses passive muscle mechanical properties and intrinsic contractile dynamics without volitional input. Consequently, ID measures muscle properties such as peak torque, power and work, whereas TMG primarily assesses various temporal characteristics of muscle contraction (Tc, Td, Tr, Ts) and muscle stiffness (Dm) (13, 18). This distinction implies that they probe different aspects of muscle function, making a direct one-to-one correlation of asymmetry measures inherently challenging.

A previous study, which revealed an inconsistent correlation between parameters of ID and TMG (19), confirms this statement. Additionally, the approach for quantifying FS and LS in ID and TMG differs, which is likely the main reason for the low and illogical correlation between methods. In ID, the equation for determining the FS implies the relationship between characteristics of knee flexor and extensor muscles (H/Q) (16). In contrast, the TMG equation for FS is more complex and may not involve all muscles of the knee joint (the ST muscle is excluded) (14), leading to a non-significant relationship in observed FS parameters between ID and TMG. Regarding LS, ID involves a comparison between limbs across all knee joint extensor/flexor muscles, while TMG measurements are focused on comparison in individual muscles (5, 14). The fact that the some significant associations are detected between methods when TMG LS parameters of all knee joint extensor/flexor muscles were combined (LS Qavg, LS Havg, Table 5 and Figure 2) further supports this statement.

The present study revealed some important insights into the comparison of the two testing approaches. As shown in Tables 3–5, a significant correlation between ID and TMG appears only in LS, with the correlation being higher for the knee joint flexor muscles and when ID is applied at higher velocities. Notably, the correlation between methods was also higher in males. The observed negative correlation between muscle asymmetry values obtained with ID and TMG was unanticipated. Given that higher values in both methods typically represent a state of greater symmetry (23, 27), a negative relationship strongly underscores that these tools are not merely measuring the same phenomenon with different scales, but rather capturing divergent aspects of muscular balance. This reinforces the conclusion that ID and TMG lack fundamental similarity in their assessment of muscle asymmetry.

The present findings indicate that ID and TMG measure different types of muscle asymmetry; they are not interchangeable, and TMG alone cannot substitute for ID in detecting lower-limb muscle asymmetries. However, TMG may serve as a complementary tool in applied settings due to its portability, efficiency, and independence from participant motivation. Practitioners should therefore consider a multi-modal approach, combining the precise strength evaluation provided by ID with the rapid, non-volitional assessment of muscle characteristics from TMG. This integrated approach can provide a more holistic understanding of an individual's muscular status, aiding in more nuanced training prescriptions, injury prevention, and rehabilitation monitoring. The observed sex-specific associations, where correlations between ID and TMG parameters were higher in males and distinct correlations existed in females, suggest that biological differences related to hormonal profiles, muscle architecture, or neuromuscular control may influence how these methodologies capture asymmetry (28, 29).

Importantly, the interpretation of the observed negative correlations requires additional methodological and physiological consideration. Although higher values in both ID and TMG symmetry indices represent greater symmetry, the use of MIN/MAX ratios to quantify asymmetry may introduce directionality artifacts, particularly when small absolute inter-limb differences are transformed into ratio-based scores (30, 31). When participants demonstrate high levels of symmetry, values tend to cluster near 1.0, potentially compressing variance and producing ceiling effects that can distort linear associations and even invert correlation direction (32). Furthermore, the physiological determinants underlying the measured variables differ substantially. TMG-derived parameters, especially Dm, predominantly reflect intrinsic muscle mechanical properties and passive stiffness characteristics (13), whereas isokinetic peak torque is strongly influenced by voluntary neural drive, motor unit recruitment, and intermuscular coordination (33, 34). Because neural drive can augment or compensate for peripheral mechanical asymmetries, joint-level torque symmetry may not directly mirror intrinsic contractile symmetry, and the relationship between these measures may therefore be non-linear or even inverse under certain conditions. Collectively, these methodological and physiological factors provide a plausible explanation for the counterintuitive negative correlations and further support the interpretation that ID and TMG capture distinct, though complementary, dimensions of neuromuscular asymmetry.

A main study strength is that it is conducted on the largest sample of participants so far, especially regarding TMG, and on a heterogeneous sample, which enables the study results’ generalisation. Study limitations should also be acknowledged. This study investigated the comparison of TMG solely with ID. Given the numerous methods for assessing muscle asymmetry, future studies should investigate the association between TMG and other dynamometry types, such as isotonic and isometric dynamometry, whose measurements, particularly isometric, are based on contractions more similar to TMG's assessment principles.

## 5 Conclusion

This study aimed to determine whether TMG can reliably detect functional and lateral asymmetries comparable to those measured by ID across sexes. Only isolated small-to-moderate associations were observed in LS, primarily sex-specific and muscle-group specific. The present findings indicate that ID and TMG measure different types of muscle asymmetry; they are not interchangeable, and TMG alone cannot substitute for ID in detecting lower-limb muscle asymmetries. For practitioners, these results emphasise the need to combine multiple assessment tools to obtain a more comprehensive picture of neuromuscular balance.

## Data Availability

The raw data supporting the conclusions of this article will be made available by the authors, without undue reservation.
